# Basic Health Insurance Package in Iran: Revision Challenges

**Published:** 2017-05

**Authors:** Reza DEHNAVIEH, Hamed RAHIMI

**Affiliations:** Health Services Management Research Center, Institute for Futures Studies in Health, Kerman University of Medical Sciences, Kerman, Iran

## Dear Editor-in-Chief

All health systems are facing a paradox of limited resources against the unlimited human needs. Therefore, any country in the world is not able to meet all the health needs of the population and provide all services. The available resource allocation in the best way possible is essential (1, 2).

Priority setting is one way for good use of limited resources (3). Priority setting in health care includes choosing among the possible options of the programs and health care services and patients or groups of patients who should receive it (4). One of the approaches that reflect the priorities selected, is to develop a Basic Health Benefit Package (BHBP). BHBP is the least services that should be available to ensure the proper condition of public health and protection against disease. Designing such a package has several benefits. With the pool of a number of interventions together, it may be possible to take advantage of common use of particular inputs and therefore reduce the costs (5).

Iran (2004) established the Ministry of Welfare and Social Security (MOWSS) to distinguish the funders and providers of health care. Since this time, health insurance organizations were under the MOWSS, and the High Council of Health Insurance (HCHI) was transferred to this ministry. This ministry was merged (2011) with two other ministries and renamed to the Ministry of Cooperatives, Labor and Social Welfare (MCLS). Iran’s health insurance system consists of numerous insurance organizations. The main insurance organizations are Social Security Organization, the Army Medical Insurance Organization, Health Insurance Organization, Imam Khomeini Relief Committee (6).

Reviewing the existing requirements in the insurance system in Iran shows that the insurance system is faced with some problems. In this regard, the formulation and revision of BHBP have always been one of the main challenges in insurance organizations of the country. In other words, these organizations are always faced with the question that, considering the dynamic of the health system, the introduction of new medicines and equipment to the health market, changing burden of disease in the course of time, and resource constraints, what process should be considered for formulation and revision of the services of this package. This question has never been answered correctly in Iranian insurance organizations.

Despite the fact that Article 10 of the Law on general insurance in Iran, referred to determination of the minimum scope and level of medical services and medications but in practice, no specific criteria and process for the review of the BHBP have not been mentioned. Documents show that since the founding Medical Service Insurance organization in 1994, the number of services covered by the insurance package has been increased. On the other hand, a process does not exist for investigating the withdrawal of services from BHBP. Lack of revision of BHBP makes the service that lose their effectiveness over time due to advances in technology, changing disease burden apply additional financial burden to the health system. The lack of updates in BHBP can be a factor in the increase in out of pocket and increase in families facing catastrophic costs.

One of the main reasons for the lack of review of the BHBP in Iran is a difficult bureaucratic process imposed by regulation on its formulation. In this process, the Ministry of Health & Medical Education (MOHME) in accordance with Article 10 provides its proposal to the HCHI. If the council agrees, the Cabinet of Ministers must approve it. During this process, the planning in the MOHME and experts’ talks in the HCHI on the one hand, and long discussions in various government commissions, on the other hand delayed the inclusion of new services to the BHBP. Despite the cumbersome process, the review of the available services in the BHBP will not be considered. In addition, the current structure of determination of the BHBP services lacks balance and sufficient competency. The combination of experts in the process is more political and a combination of professional and academic people is less used. For example, the combination of HCHI members has been formed of ministers, representatives of insurance organizations and Members of Parliament. The HCHI does not have expert power for scientific analysis and decision making for inclusion or exclusion of the services from the BHBP. Another challenge for BHBP is low participation of some groups, including the private sector, public, NGOs and health economists.

Thus, it is essential to Iran’s policy makers to design and implement an evidence-based process for reviewing the substance of BHBP. In the design of the process of formulating and reviewing the package two important points should be noted, the use of professional experts and specialists, and the use of scientific tools and metrics to assess the services such as Health Technology Assessment.
